# Morphology, Composition, and Bioactivity of Strontium-Doped Brushite Coatings Deposited on Titanium Implants via Electrochemical Deposition

**DOI:** 10.3390/ijms15069952

**Published:** 2014-06-04

**Authors:** Yongqiang Liang, Haoyan Li, Jiang Xu, Xin Li, Mengchun Qi, Min Hu

**Affiliations:** 1 Department of Stomatology, Chinese PLA General Hospital, Beijing 100853, China; E-Mail: lych810@foxmail.com; 2 College of Stomatology, Hebei United University, Tangshan 063000, China; E-Mails: dingding627@foxmail.com (H.L.); lych810@163.com (X.L.); haiyang156@foxmail.com (M.Q.); 3 Department of stomatology, Tongchuan City People’s Hospital, Tongchuan 727000, China; E-Mail: xujiang168@hotmail.com

**Keywords:** strontium, brushite, coating, implant, osteoblast, biomedical materials

## Abstract

Surface modification techniques have been applied to generate titanium implant surfaces that promote osseointegration for use in dental applications. In this study, strontium-doped brushite coatings were deposited on titanium by electrochemical deposition. The phase composition of the coating was investigated by energy dispersive X-ray spectroscopy and X-ray diffraction. The surface morphologies of the coatings were studied through scanning electron microscopy, and the cytocompatibility and bioactivity of the strontium-doped brushite coatings were evaluated using cultured osteoblasts. Osteoblast proliferation was enhanced by the addition of strontium, suggesting a possible mechanism by which strontium incorporation in brushite coatings increased bone formation surrounding the implants. Cell growth was also strongly influenced by the composition of the deposited coatings, with a 10% Sr-doped brushite coating inducing the greatest amount of bone formation among the tested materials.

## 1. Introduction

Early osseointegration is critical for the clinical success of oral implants. Therefore, the appropriate geometry and surface topography of implant materials, which can greatly influence osseointegration, are crucial for both the short- and long-term success of dental implants. As an inert material, titanium (Ti) exhibits good biocompatibility, good corrosion resistance, high strength, and low elastic modulus. For these reasons, titanium has become the most widely used material in the fabrication of oral implants. However, the bio-inert nature of Ti and Ti alloy metal also limits the integration of implant surfaces with surrounding bone tissue. To strengthen the interface between Ti implants and bone, many studies have focused on improving the surface properties of Ti implant materials, with the goal of achieving better osseointegration in less time.

One approach to modifying the surface properties of Ti is through application of a coating made of a biologically active material. The fast bone regeneration (FBR) coating (Pitt-Easy, Oraltronics, Bremen, Germany) is composed of fully resorbable calcium phosphate (CaP) prepared from brushite via electrochemical deposition on Ti plasma-sprayed (TPS) implants [1]. The bioactive CaP layer is completely resorbed within 6–12 weeks after implantation, and upon resorption, newly formed bone is in direct contact with the roughness of the TPS surface [2]. Brushite, which is also known as dicalcium phosphate dihydrate (CaHPO_4_·2H_2_O), is characterized by a 1:1 Ca:P ratio. It is stable in weakly acidic environments of pH 4–6 [3] and has been shown to be both biocompatible and osteoconductive, indicating that this coating can promote implant osseointegration and bone formation. *In vitro* animal experiments have demonstrated the potential utility of brushite in bone tissue engineering [4]. In addition, one study reported that a resorbable brushite coating is bioactive because relative bone growth on brushite-coated Ti (48.8%) was greater than that on pure Ti controls (22.4%) [5].

Strontium (Sr) is an essential trace element in human skeletal components. A previous study confirmed that the effects of Sr and calcium on metabolic behavior are almost identical in the skeletal system. Sr can promote bone formation and inhibit bone absorption [6]. In both *in vitro* and *in vivo* tests, Sr promotes osteoblast proliferation and inhibits osteoclast proliferation, and *in vitro* animal experiments have shown that Sr positively affects bone regeneration [7].

In the present study, we used an electrochemical deposition method to prepare Sr-doped brushite coatings on Ti. We hypothesized that a synergistic effect of brushite and Sr in Ti coatings can enhance the osseointegration efficiency of implants, thereby supporting the development of novel and more successful dental implants.

## 2. Results

### 2.1. Phase Composition of Sr-Doped Brushite Coatings

**Figure 1:**
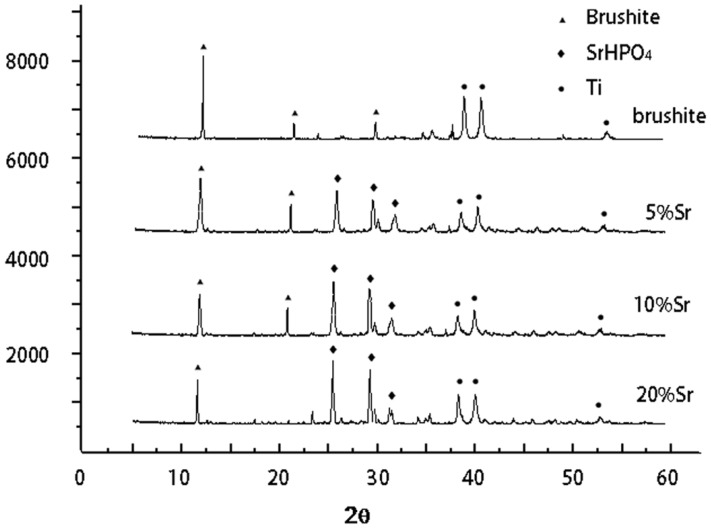
XRD analysis in brushite, 5% Sr, 10% Sr and 20% Sr groups.

XRD analysis demonstrated the phase composition of the pure and Sr-doped brushite coatings. The relative peak intensities of the diffraction peaks for brushite crystals (020) in the brushite group were higher than those of other surface crystal types, indicating that crystal growth occurred along the (020) preferred orientation of the crystal face. The relative peak intensities of the diffraction peaks for Sr-doped crystals (102) in the SrHPO_4_-doped groups were higher than those of other diffraction peaks, as shown in (102) crystal face growth advantage. The diffraction peak intensity of SrHPO_4_ peaks was increased in the 5%, 10%, and 20% Sr groups, indicating a growth advantage of SrHPO_4_ crystals (102). The relative peak diffraction of brushite crystals (020) in the 20% group was slightly lower, which may be because the increasing amount of Sr restrained the formation of brushite Figure 1.

### 2.2. Morphology of Sr-Doped Brushite Coatings

**Figure 2:**
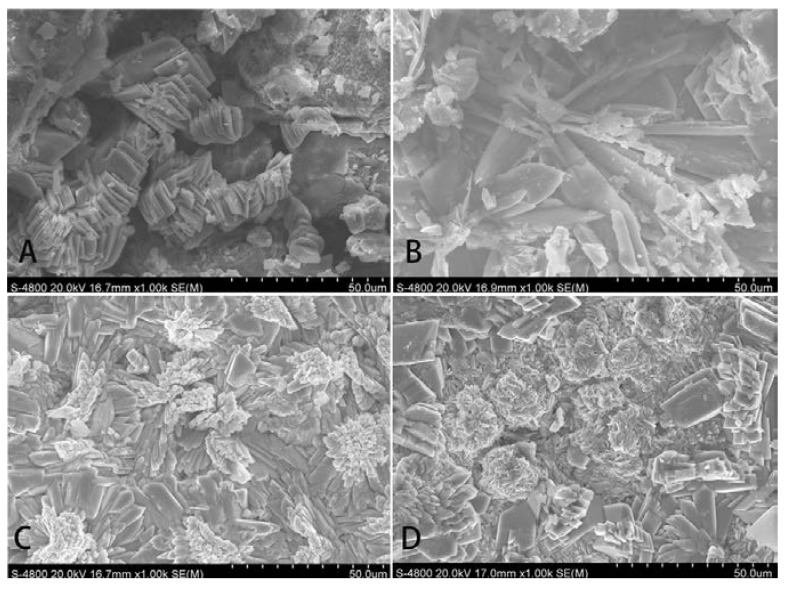
Surface micro topography by SEM analysis: (**A**) Brushite group; (**B**) 5% Sr group; (**C**) 10% Sr group; (**D**) 20% Sr group.

According to SEM analysis, the average thickness of Sr-doped brushite coatings was 15–35 µm in the cross-sectional samples. SEM images also demonstrated that the coating consisted of lamellar crystals of different size and shape. The brushite coating contained larger crystals, some of which were arranged in clusters of divergents and others that were in a disordered arrangement. Flakey crystals were observed in the brushite coating containing 5% Sr. The crystals were fine and compact at the top, but wide and loose at the bottom. The brushite coating containing 10% Sr included two different sizes of crystals, smaller lamellar crystals arranged in clusters and larger lamellar crystals in a petal-shaped arrangement. The brushite coating containing 20% Sr showed an irregular surface morphology with a clustering sheet structure and other structural irregularities Figure 2.

### 2.3. Elemental Analysis of Sr-Doped Brushite Coatings

**Figure 3:**
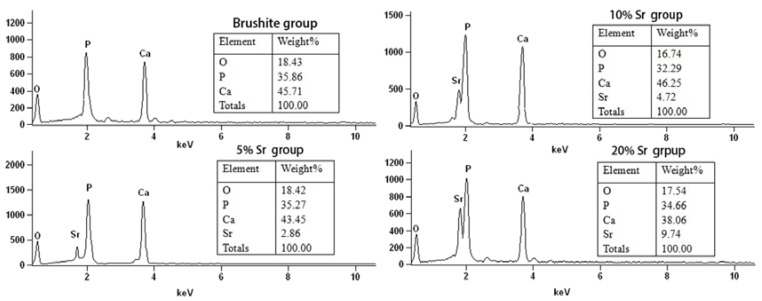
Coating element analysis using energy dispersive X-ray spectroscopy (EDS).

The EDS results showed that the atomic ratio of Ca to P was roughly 1.1:1. The coating surface was rich in calcium and phosphates. EDS analysis confirmed the presence of brushite and SrHPO_4_ on the coating surfaces Figure 3.

### 2.4. Microarchitecture of Bone Formed Around Sr-Doped Brushite-Coated Ti Implants

**Figure 4:**
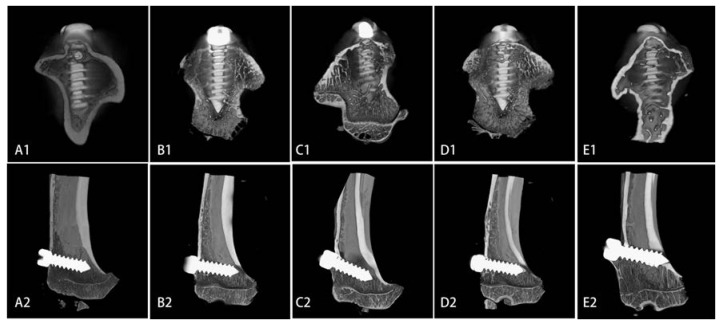
Micro-computed tomography (Micro-CT) images of the proximal tibia with implants in (**A**) Ti group; (**B**) brushite group; (**C**) 5% Sr group; (**D**) 10% Sr group; (**E**) 20% Sr group; **1**: vertical axis; **2**: long axis.

High-resolution, three-dimensional (3-D) images obtained using micro-CT clearly depicted differences among the five groups Figure 4. As expected, the Ti group showed the lowest values in IBCR (55.76%), BV/TV (52.77%), and the associated morphological parameters such as Conn.D (103.70 mm^−3^), Tb.Th (0.1621 mm), Tb.Sp (0.2117 mm), and Tb.N (5.44 mm^−1^). The 5% and 10% Sr-doped brushite coatings were associated with increased 3-D bone volume, and most of the bone parameters for the 10% Sr coatings were comparable to those of the Ti, brushite-coated, and 20% Sr-doped brushite coated implants (*p* < 0.05). More bone formation was observed around the 10% Sr-doped brushite-coated implants Table 1.

**Table 1:** Micro-CT analysis of bone indices among five groups (*n* = 12).

Indices	Ti	Brushite	5% Sr	10% Sr	20% Sr
IBCR	55.76 ± 3.95	60.47 ± 3.97	61.99 ± 3.55 *	67.43 ± 3.53 *^Δ■^	63.04 ± 4.65 *
BV/TV (%)	52.77 ± 6.81	54.22 ± 5.72	57.12 ± 6.78 *	65.43 ± 4.68 *^Δ■^	58.19 ± 5.62
Conn.D	103.70 ± 6.25	109.45 ± 8.90	114.57 ± 8.94 *	137.42 ± 6.76 *^Δ■^	117.51 ± 7.01 *^□^
Tb.Th	0.1621 ± 0.0100	0.1796 ± 0.0111 *^■^	0.2077 ± 0.0226 *	0.2089 ± 0.0188 *	0.1719 ± 0.0124 ^■□^
Tb.Sp	0.2117 ± 0.0071	0.2206 ± 0.0053 *	0.3241 ± 0.03 *^Δ^	0.5694 ± 0.3293 *^■^	0.3133 ± 0.0119 *^□^
Tb.N	5.44 ± 0.28	7.63 ± 0.70 *	7.98 ± 0.80 *	8.96 ± 0.64 *^Δ■^	6.85 ± 0.62 *^■□^

* *p* < 0.05 comparing with the Ti group; ^Δ^ *p* < 0.01 comparing with the brushite group; ^■^ *p* < 0.05 comparing with the 5% Sr group; ^□^ *p* < 0.05 comparing with the 10% Sr group.

### 2.5. Biomechanical Strength of Sr-Doped Brushite-Coated Implants

The measured values for removal torque were 27.11 ± 3.37, 28.12 ± 2.52, 32.30 ± 2.17, 33.30 ± 2.17, and 26.30 ± 2.17 N.cm for the pure Ti, brushite-coated, 5% Sr-doped brushite-coated, 10% Sr-doped brushite-coated, and 20% Sr-doped brushite-coated implants, respectively. Removal torque in 5% Sr and 10% Sr groups was significantly increased when compared with the other three groups (*p* < 0.01).

### 2.6. Cytotoxicity of Sr-Doped Brushite Coatings on Ti Implants

The cell toxicity ratings for the five experimental brushite coatings were 0 or 1. The coating in all five groups induced no toxicity to MC3T3-E1 cells (*p* > 0.05). After MC3T3-E1 cell culture for 24, 48, or 72 h in medium containing extracts of the different coatings, the numbers of proliferating cells in the experimental groups (brushite-coated and Sr-doped brushite coated) were higher than that in the control Ti group (*p* < 0.01). With an increasing Sr content from 5% to 10%, the number of MC3T3-E1 cells significantly increased, and the largest number of MC3T3-E1 cells was observed for the 10% Sr-doped coating. The number of MC3T3-E1 cells observed after exposure to extract of the 20% Sr-doped brushite coating was significantly less than the results for the 5% Sr- and 10% Sr-doped brushite coatings (*p* < 0.01; Table 2).

**Table 2:** OD value of osteoblast cultured in the Sr-brushite extracted fluid (*n* = 6).

Groups	24 h	48 h	72 h
Ti	0.3126 ± 0.0113	0.4956 ± 0.0082	0.5725 ± 0.0170
brushite	0.4133 ± 0.0183 *	0.5153 ± 0.0109 *	0.6155 ± 0.0125 *
5% Sr	0.4782 ± 0.0188 *^Δ^	0.5873 ± 0.0099 *^Δ^	0.6890 ± 0.0128 *^Δ^
10% Sr	0.5119 ± 0.0402 *^Δ^	0.6193 ± 0.0315 *^Δ■^	0.7561 ± 0.0582 *^Δ■^
20% Sr	0.4222 ± 0.0136 *	0.5278 ± 0.0139 *	0.6180 ± 0.0270 *

* *p* < 0.01 comparing with the Ti group; ^Δ^ *p* < 0.01 comparing with the brushite and 20% Sr group; ^■^ *p* < 0.05 comparing with the brushite and 5% Sr group.

## 3. Discussion

The surface characteristics of dental implants play an important role in the osseointegration process. Surface treatments that have been applied to modifying Ti surfaces include plasma spraying, coating, sandblasting, acid etching, anodizition, wet chemical methods, hydrothermal synthesis, the sol-gel method, biomimetic synthesis, and electrochemical deposition. Among these, the electrochemical deposition method can be used to produce porous coatings in the basement complex at low reaction temperature through simple processing [8], and thus, this method represents a biological coating preparation technology with wide application potential.

In the present study, Sr-doped brushite coatings with different microstructures and morphologies were successfully produced by electrochemical deposition. The morphology and composition of electrodeposited, Sr-doped brushite coatings could be controlled by adjusting the processing parameters. The results confirm that simple conditions of an experimental electrolysis voltage of 2.5 V and reaction temperature of 60 °C afforded easy formation of Sr-doped brushite coatings on Ti implants. The Sr content of the brushite coating can be easily controlled by adjusting the Sr concentration during electrochemical deposition. A small pore structure between tiny crystals ensures that the electrical conductivity of the coatings on the metal base was not interrupted, allowing the deposition process to continue [9]. In the present study, we made use of this pore structure by depositing SrHPO_4_ in the brushite crystals pores, or brushite in the SrHPO_4_ pores. The surface of implants showed more microporous after acid and alkali treatment, which increased the mechanical interlock and attachment between the coatings and the core titanium. In this experiment, simple scratch tester verified the Sr-doped brushite coatings had certain binding strength. There was no coating deattachment during implantation. In future experiment, we hope to explore the relation between absorption velocity of coating and new bone formation through observing the sections of hard tissues containing implants with Sr-doped brushite coatings.

Sr can reduce bone resorption by inhibiting osteoclast activity and improve bone formation by stimulating osteoblast activity [10,11,12]. Specifically, Sr can promote osteogenic cell replication and osteoblast activity, including bone matrix synthesis and alkaline phosphatase production. By contrast, Sr can reduce osteoclast markers generated during bone marrow cell differentiation, inhibit osteoclast differentiation, and reduce osteoclast activity [13,14]. Moreover, Sr has been shown to stimulate osteogenic differentiation of bone marrow mesenchymal stem cells and other progenitor cells [15,16]. Finally, multiple studies have shown that Sr-doped hydroxyapatite, calcium phosphate, calcium silicate, calcium sulfate, boron bioactive glass, and other materials promote bone tissue reconstruction and new bone formation [17,18,19,20].

With the development of advanced imaging technologies, quantitative CT and micro-CT have significantly increased capabilities for evaluation of bone structure parameters [21,22]. In the present study, the following microarchitectural parameters around implants were assessed in micro-CT images of VOIs: IBCR, BV/TV, Conn.D, Tb.Th, Tb.Sp, and Tb.N. The results showed that Sr-doped brushite coatings can improve implant osseointegration, and the 10% Sr coating exhibited the best properties for implant osseointegration among the tested coatings.

In addition to the micro-CT results, mechanical removal tests also demonstrated that Sr-doped brushite coatings enhanced the osseointegration of Ti implants. The bioactive properties of these coatings were demonstrated by bone growth on coated Ti implants, which was higher than that on noncoated implants.

Cell proliferation is highly dependent on the chemical composition, topography, and surface roughness of the materials. In the electrochemically deposited coatings, doping with Sr showed a positive effect on the proliferation of MC3T3-E1 cells. The cell responses to the coatings may be attributed to a combination of influences from the Sr dose. In 10% Sr-doped coating group significantly higher bone formation and higher cell proliferation were observed. Meanwhile in 20% group bone formation and cell proliferation decreased comparing with 10% group. The increasing amount of Sr in 20% group may inhibit the osteoblasts activity.

In conclusion, a 10% Sr-doped brushite coating can promote implant osseointegration and also increased the proliferation of osteoblasts.

## 4. Materials and Methods

### 4.1. Preparation of Sr-Doped Brushite Coating by Electrochemical Deposition

Commercial Grade 4 unalloyed titanium plates (Baoji Titanium Industry Company Limited, Baoji, China) with dimensions of 10 × 10 × 2 mm were used as substrates for electrochemical deposition. Before deposition of the coating, the titanium plates were polished to 2000 grid, immersed in hydrochloric acid and calcium chloride solutions successively. Then they were ultrasonically cleaned in acetone for 10 min, rinsed with deionized water, and air dried. The experimental set-up for electrochemical deposition used in this study consisted of a simple two-electrode cell configuration [23]. The working electrode (the coating substrate) was the titanium alloy plate, and the counter electrode was a platinum mesh. The distance between the electrodes was 2 cm, and a constant voltage of 2.5 V was applied. The temperature was 60 °C, and the reaction time was 1 h. The electrolyte solution composition was 0.036 M NH_4_H_2_PO_4_, 0.06 M CaCl_2_ and SrCL_2_, and 0.2 M NaCl_2_, pH 4.5. Electrochemical deposition was used to apply coatings composed of pure brushite and brushite containing 5%, 10%, and 20% Sr, depending on the molar ratio of Sr and Ca [Sr/(Sr + Ca) = 0%, 5%, 10%, or 20%]. Thus, the final experimental groups included Ti samples coated with four different proportions of Sr in the brushite and pure brushite for comparison.

### 4.2. Morphology and Characterization of Sr-Doped Brushite Coatings

The phase composition of the coating material was analyzed by X-ray diffraction (XRD) (D/MAX2500PC, Rigaku, Tokyo, Japan). The surface morphology and coating thickness were examined by scanning electron microscopy (SEM) using a Hitachi S-4800 instrument (Hitachi, Tokyo, Japan). The elemental composition of the biomimetic coatings was determined using SEM was coupled with energy dispersive X-ray spectroscopy (EDS, Noran7, Thermo Fisher, Waltham, MA, USA).

### 4.3. Preparation of Ti Implants Coated with Sr-Doped Brushite

The Ti implants were custom-made, screw-type Ti implants (Ø1.5 × 6.5mm, 99.8% Ti) prepared at the Engineering Research Center in Biomaterials of Sichuan University. For electrochemical deposition of Sr-doped brushite coatings, the Ti implants were treated using the procedure described above for Ti plates. The experimental groups included implants coated with pure brushite and brushite containing 5% Sr, 10% Sr, and 20% Sr, according to the molar ratio of Sr and Ca [Sr/(Sr + Ca) = 0%, 5%, 10%, and 20%]. Pure Ti implants were used as the control.

### 4.4. Implantation of Brushite-Coated Ti Implants

Sixty 12-week-old adult female Sprague–Dawley (SD) rats were used in this study. All experimental procedures were approved by the Animal Care and Use Committee of Hebei United University [No. SCXK (Jun 2009-003)] and conducted in accordance with the guidelines for the care and use of laboratory animals.

Twenty four implants with 5% Sr-doped brushite coatings, 24 implants with 10% Sr coatings, 24 implants with 20% Sr coatings and 24 pure Ti implants were prepared. These 120 implants were sterilized by high-pressure sterilization method. After 1 week of acclimatization, all rats underwent bilateral insertion of implants in the proximal metaphysis of tibiae under general anesthesia. After 10 mm incisions were made at the bilateral proximal metaphysis of the tibiae, holes were drilled for implant insertion. Then the implants were screwed into the holes, and the soft tissues were sutured in layers. Analgesia and prophylactic antibiotics were also administrated to animals.

### 4.5. Micro-Computed Tomography (Micro-CT) Examination

At 8 weeks after implantation, rats tibiae with implants were harvested and one tibia of each rat was placed in a custom container with water, and scanned by a micro-computed tomography (CT) 80 scanner (Scanco Medical, Bassersdorf, Switzerland) in an axial direction vertical to the long axis of the implant [24]. Another tibia of each rat was saved for future hard tissue slicing. In total, 10 mm of each proximal tibia was examined. The volume of interest (VOI) was selected around the implant and defined as a column from the implant axis with a radius of 3.0 mm. The following microarchitectural parameters were assessed in VOI images: implant-bone contact rate (IBCR, %), bone volume ratio (BV/TV, %), connective tissue density (Conn.D, mm^−3^), trabecular thickness (Tb.Th, mm), trabecular separation (Tb.Sp, mm), and trabecular number (Tb.N, mm^−1^).

### 4.6. Biomechanical Testing of Brushite-Coated Ti

The removal torque for the implants was examined as previously described [24]. Briefly, the specimens were fixed in 10% neutral buffered formalin and embedded in dental plaster. The equipment used for biomechanical testing included a force measurer (DZE-5, Asida, Zhengye Electronics, Dongguan, China) for recording the maximum force (N) required to loosen the implant and a custom wrench that was used to connect the implant at one end and the force measurer at the other end. The implants used were specially designed with a square cap for holding the wrench. The removal torque was calculated by multiplying the maximum force value with the distance between the force point and the center of the implant.

### 4.7. MTT Assay for Cytotoxicity of Brushite-Coated Ti

The five groups of Ti plates (pure brushite and brushite containing 5%, 10%, or 20% Sr) were immersed in culture medium and then allowed to stand for 72 h at 37 °C in an aseptic environment to prepare the extract-containing fluid. A suspension of 1 × 10^4^ cells/mL MC3T3-E1 osteoblasts (purchased from the Medical Cell Center, Institute of Basic Medical Sciences, Chinese Academy of Medical Sciences) was prepared and cultured in the extract-containing fluid. The absorption value was measured using an ultraviolet spectrophotometer (photoLab 6600 UV-VIS, Munich, Germany) after 24, 48, and 72 h of culture in the extract-containing fluid. As an indicator of cytotoxicity, the relative growth rates (RGRs) were calculated: RGR (%) = average absorbance value (OD value) of experimental group/OD value of control group × 100%. Cell toxicity grading: grade 0 ≥ 100%, grade 1 = 80%–99%, grade 2 = 50%–79%, grade 3 = 30%–49%; and grade 4 = 0%–29%.

### 4.8. Statistical Analysis

Data are expressed as mean ± standard deviation (SD), and statistical analyses were performed using SPSS 12.0 (SPSS Inc., Chicago, IL, USA). One-way analysis of variance was conducted to assess differences between all quantitative indices among the three groups, and the Dunnett T3 method was applied for multiple comparisons. *p* < 0.05 was considered to be significantly different.

## 5. Conclusions

In conclusion, the addition of strontium leads to enhanced proliferation, suggesting the possible benefits of strontium incorporation in brushite coatings. The composition of deposited coatings showed a strong influence on the growth of cells. The 10% strontium coating has a promoting effect on implant osseointegration and also increased the proliferation of osteoblasts.
